# Supplementary material for: Machine Learning Model Based on Insulin Resistance Metagenes Underpins Genetic Basis of Type 2 Diabetes
Source: Biomolecules. 2023 Feb 24;13(3):432. doi: 10.3390/biom13030432 (PMC10046262; doi:10.3390/biom13030432)
Supplement: Supplementary file 1 [file biomolecules-13-00432-s001.zip › biomolecules-2186728-supplementary.pdf]

List of 72 Key meta-genes

S. No.	Gene Symbol	GeneName	Entrez Gene
1	ACTA2	actin alpha 2, smooth muscle	59
2	ALDH6A1	aldehyde dehydrogenase 6 family member A1	4329
3	AMPD3	adenosine monophosphate deaminase 3	272
4	APOB	apolipoprotein B	338
5	ARID5B	AT-rich interaction domain 5B	84159
6	ATF6B	activating transcription factor 6 beta	1388
7	ATP2B2	ATPase plasma membrane Ca2+ transporting 2	491
8	ATXN1	ataxin 1	6310
9	B2M	beta-2-microglobulin	567
10	BHLHE40	basic helix-loop-helix family member e40	8553
11	BICD1	BICD cargo adaptor 1	636
12	CASQ2	calsequestrin 2	845
13	CAT	catalase	847
14	CCL19	C-C motif chemokine ligand 19	6363
15	CD24	CD24 molecule	100133941
16	CEP350	centrosomal protein 350	9857
17	CFB	complement factor B	629
18	CHD2	chromodomain helicase DNA binding protein 2	1106
19	CHI3L1	chitinase 3 like 1	1116
20	COL8A1	collagen type VIII alpha 1 chain	1295
21	CROCC	ciliary rootlet coiled-coil, rootletin	9696
22	DCAF8	DDB1 and CUL4 associated factor 8	50717
23	DDX17	DEAD-box helicase 17	10521
24	EIF2AK2	eukaryotic translation initiation factor 2 alpha kinase 2	5610
25	EIF4G3	eukaryotic translation initiation factor 4 gamma 3	8672
26	EMX2	empty spiracles homeobox 2	2018
27	ERF	ETS2 repressor factor	2077
28	FAM98A	family with sequence similarity 98 member A	25940
29	FHL2	four and a half LIM domains 2	2274
30	FOXF2	forkhead box F2	2295
31	GAS2	growth arrest specific 2	2620
32	GATM	glycine amidinotransferase	2628
33	HDAC4	histone deacetylase 4	9759
34	HIPK1	homeodomain interacting protein kinase 1	204851
35	HIPK3	homeodomain interacting protein kinase 3	10114
36	HMMR	hyaluronan mediated motility receptor	3161
37	HOMER1	homer scaffold protein 1	9456
38	HS3ST1	heparan sulfate-glucosamine 3-sulfotransferase 1	9957
39	IGFBP5	insulin like growth factor binding protein 5	3488
40	INSR	insulin receptor	3643
41	IRX5	iroquois homeobox 5	10265
42	LSM3	LSM3 homolog, U6 small nuclear RNA and mRNA degradation associated	27258
43	MAF	MAF bZIP transcription factor	4094

44	MAP3K5	mitogen-activated protein kinase kinase kinase 5	4217
45	MAPK6	mitogen-activated protein kinase 6	5597
46	MARCO	macrophage receptor with collagenous structure	8685
47	MATK	megakaryocyte-associated tyrosine kinase	4145
48	MMP9	matrix metallopeptidase 9	4318
49	MYL12A	myosin light chain 12A	10627
50	NDUFB8	NADH:ubiquinone oxidoreductase subunit B8	4714
51	NDUFS1	NADH:ubiquinone oxidoreductase core subunit S1	4719
52	NRCAM	neuronal cell adhesion molecule	4897
53	NRGN	neurogranin	4900
54	PFDN4	prefoldin subunit 4	5203
55	PHKB	phosphorylase kinase regulatory subunit beta	5257
56	PRSS3	serine protease 3	5646
57	HBD	hemoglobin subunit delta	3045
58	RASSF7	Ras association domain family member 7	8045
59	REV3L	REV3 like, DNA directed polymerase zeta catalytic subunit	5980
60	RPS15A	ribosomal protein S15a	6210
61	RPS27	ribosomal protein S27	6232
62	S100P	S100 calcium binding protein P	6286
63	SDHB	succinate dehydrogenase complex iron sulfur subunit B	6390
64	SLC19A2	solute carrier family 19 member 2	10560
65	STK24	serine/threonine kinase 24	8428
66	TAF6L	TATA-box binding protein associated factor 6 like	10629
67	UQCRC2	ubiquinol-cytochrome c reductase core protein 2	7385
68	UROD	uroporphyrinogen decarboxylase	7389
69	VSIG4	V-set and immunoglobulin domain containing 4	11326
70	WAS	Wiskott-Aldrich syndrome	7454
71	WNT4	Wnt family member 4	54361
72	ZEB1	zinc finger E-box binding homeobox 1	6935

Details of ML methods and tuning parameters

The values of Penalty Coefficient C were set to 0.001 for LASSO and 0.2 for SVM (with radial kernel). For XGBoost, the learning rate was 0.1 and the value of C was 0.5.

The number of nodes in input layers were set as per the number of key meta-genes selected by LASSO. Three hidden layers with bias initializer 1 were used containing 154, 128, and 64 neurons respectively. First two hidden layers had linear activation function and last hidden layer had ReLU activation function. An output layer with sigmoid activation function was used for prediction. Schematics of proposed ANN is shown in figure 1. All the models were implemented in python using its library scikit-learn in jupyter notebooks.

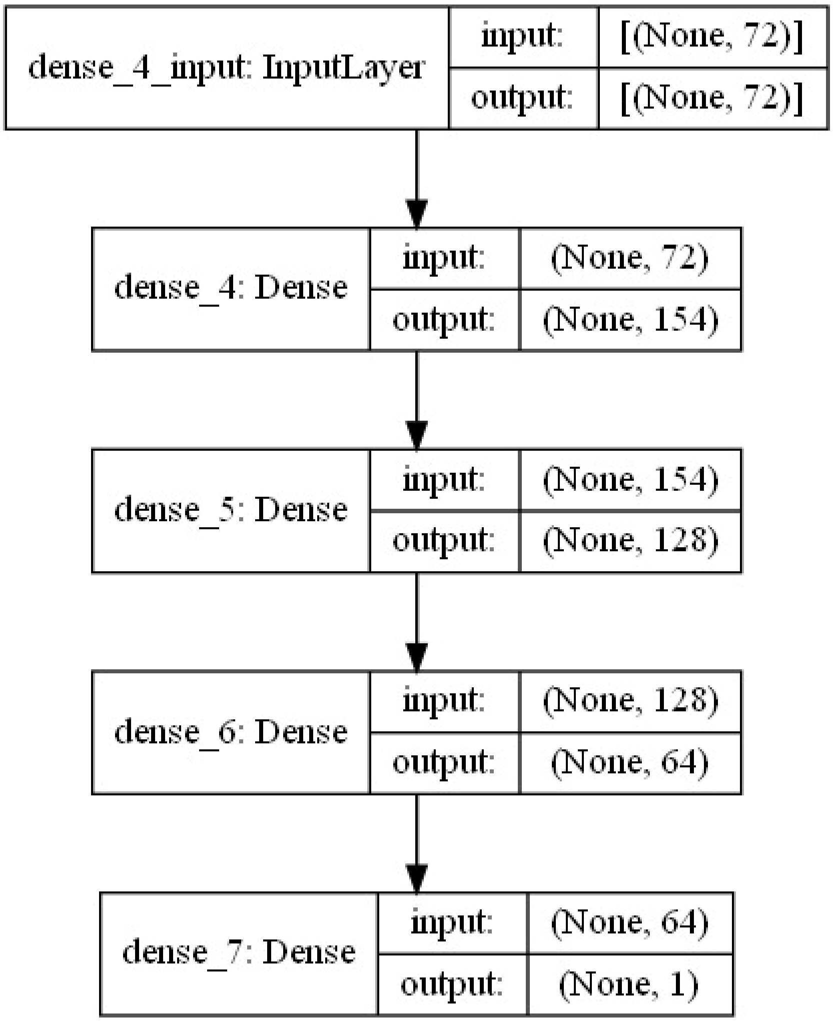

Figure 1. Architecture of proposed Artificial Neural Network
